# Correction: Value evaluation of serum (sdLDLc*HCYc)/HDLc ratio in the stability of intracranial arterial plaques in patients with acute cerebral infarction

**DOI:** 10.3389/abp.2026.17388

**Published:** 2026-07-29

**Authors:** Hongyu Hao, Xing Xing, Yajing Li, Hongshan Chu, Lei Zhao, Siqi Cheng, Yang Liu, Tiankui Wang, Nan Meng, Ruisheng Duan

**Affiliations:** 1 Department of Neurology, Hebei General Hospital, Shijiazhuang, China; 2 Hebei Provincial Key Laboratory of Cerebral Networks and Cognitive Disorders, Shijiazhuang, China; 3 Department of Pharmacy, Hebei General Hospital, Shijiazhuang, China; 4 Department of Pathology, Hebei General Hospital, Shijiazhuang, China; 5 Department of Medical Service, Hebei General Hospital, Shijiazhuang, China

**Keywords:** sdLDLc, HDLc, HCYc, acute cerebral infarction, stability of intracranial arterial plaques

The funder the Medical Science Research Project of Hebei (20200022) was erroneously omitted.

The correct statement reads:

“Acknowledgements of Financial Support: This study was funded by the Government-funded clinical excellence program (NO. ZF2023186) and the Medical Science Research Project of Hebei (20200022).”

